# High-resolution photoelectron imaging and resonant photoelectron spectroscopy *via* noncovalently bound excited states of cryogenically cooled anions

**DOI:** 10.1039/c9sc03861b

**Published:** 2019-09-16

**Authors:** Guo-Zhu Zhu, Lai-Sheng Wang

**Affiliations:** a Department of Chemistry , Brown University , Providence , RI 02912 , USA . Email: Lai-Sheng_Wang@brown.edu

## Abstract

Valence-bound anions with polar neutral cores (*μ* > ∼2.5 D) can support dipole-bound excited states below the detachment threshold. These dipole-bound states (DBSs) are highly diffuse and the weakly bound electron in the DBS can be readily autodetached *via* vibronic coupling. Excited DBSs can be observed in photodetachment spectroscopy using a tunable laser. Tuning the detachment laser to above-threshold vibrational resonances yields vibrationally enhanced resonant photoelectron spectra, which are highly non-Franck–Condon with much richer vibrational information. This perspective describes recent advances in the studies of excited DBSs of cryogenically cooled anions using high-resolution photoelectron imaging (PEI) and resonant photoelectron spectroscopy (rPES). The basic features of dipole-bound excited states and highly non-Franck–Condon resonant photoelectron spectra will be discussed. The power of rPES to yield rich vibrational information beyond conventional PES will be highlighted, especially for low-frequency and Franck–Condon-inactive vibrational modes, which are otherwise not accessible from non-resonant conventional PES. Mode-selectivity and intra-molecular rescattering have been observed during the vibrationally induced autodetachment. Conformer-specific rPES is possible due to the different dipole-bound excited states of molecular conformers with polar neutral cores. For molecules with *μ* ≪ 2.5 D or without dipole moments, but large quadrupole moments, excited quadrupole-bound states can exist, which can also be used to conduct rPES.

## Abstract 2

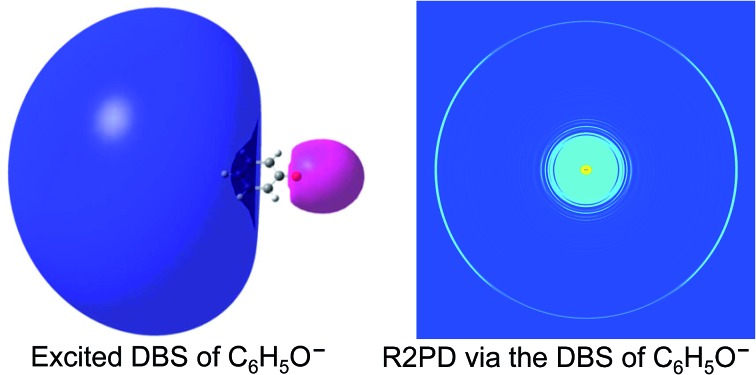
Noncovalently bound excited states of anions have led to the development of resonant photoelectron spectroscopy with rich vibrational and dynamical information.

## 1 Introduction

When a neutral molecule possesses a large dipole moment (*μ* > ∼2.5 D), it can bind an excess electron because of the long-range charge–dipole interaction with a binding energy on the order of a few to few hundreds meV.^1^–^3^ Valence-bound anions with polar neutral cores can support an excited dipole-bound state (DBS) with a diffuse orbital, analogous to Rydberg states in neutral molecules. Dipole-bound anions constitute a class of many-body systems to study electron–molecule interactions, such as vibronic coupling^4^ and low-energy electron rescattering.^5^ DBSs have been proposed as the “doorway” to the formation of stable valence-bound anions,^6^–^8^ especially for those formed in the DNA damage process by low-energy electron attachment^9^ and those in the interstellar medium under astronomical environments.^10^

Fermi and Teller first predicted a minimum dipole moment of 1.625 D for a finite dipole to bind an electron when studying the capture of negative mesotrons in 1947.^11^ Subsequently, many theoretical groups obtained a similar value of minimum dipole moment for finite dipoles to bind an electron, which was discussed by Turner in an interesting historical perspective.^12^ Further theoretical calculations showed that the critical dipole moment for electron-binding could be up to 2.0 D by including molecular effects, such as molecular rotation, moment of inertia, and dipole length.^13^–^15^ A more practical critical dipole moment of ∼2.5 D was suggested empirically from experimental observations.^1^,^16^ More recently, theoretical attention has been focused on the electron binding energies in dipole-bound anions, the nature of the electron–molecule interactions in DBSs, and the transition from DBSs to valence-bound anions.^17^–^25^

Direct evidence of DBSs came from photodetachment experiments of the enolate anion, which revealed sharp peaks in the photodetachment spectra attributed to the existence of dipole-supported excited states.^26^,^27^ Subsequently, high-resolution photodetachment spectroscopy (PDS) for a series of anions was performed to investigate rotational autodetachment *via* excited DBSs.^28^–^31^ In addition to studies of dipole-bound excited states of valence-bound anions, there have been major experimental efforts for ground-state dipole-bound anions for neutral molecules that cannot form stable valence-bound anions. A variety of dipole-bound anions were successfully produced by Rydberg electron transfer^1^,^7^,^16^,^32^–^37^ to dipolar molecules or clusters, which did not form valence-bound anions. In addition, the dynamics of DBSs of anionic clusters and complexes have been studied by time-resolved photoelectron spectroscopy (PES).^9^,^38^–^40^

**Fig. 1:**
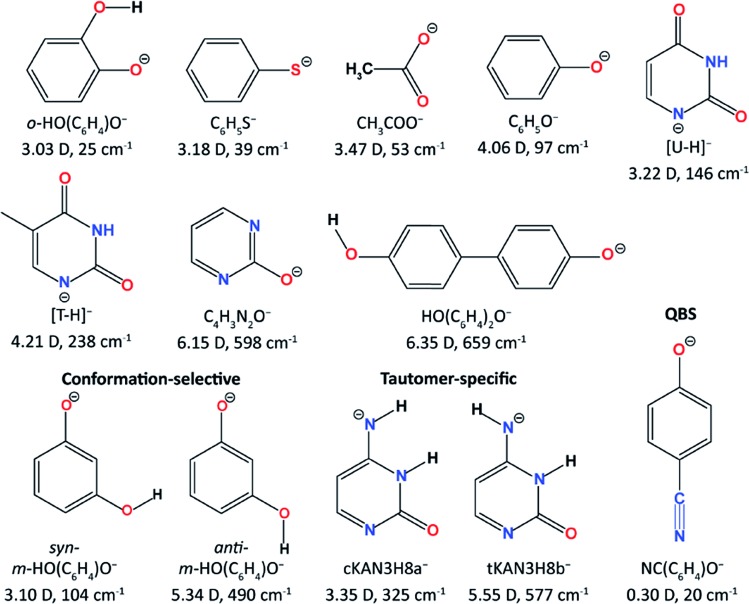
The molecular structures of all the studied cryogenically cooled anions. The dipole moments of the corresponding neutrals and the measured binding energies of the DBSs are given below the molecular structures.

The Wang group first reported high-resolution rPES *via* vibrational autodetachment from dipole-bound excited states of cryogenically cooled C_6_H_5_O^–^.^41^ The DBS of C_6_H_5_O^–^ was found to be 97 cm^–1^ below the detachment threshold. Mode-specific autodetachment from eight vibrational levels of the DBS was observed, yielding highly non-Franck–Condon resonant photoelectron (PE) spectra, due to the Δ*v* = –1 vibrational propensity rule.^42^,^43^ Subsequently, more deprotonated organic molecular anions (Fig. 1) were found to support excited DBSs^44^–^53^ or quadrupole-bound states (QBSs)^54^ below the anion photodetachment threshold. As shown in Fig. 1, DBS binding energies for various anions were measured, ranging from 25 cm^–1^ to 659 cm^–1^ depending on the dipole moments of the neutral cores. The small binding energies confirm the weakly bound nature of the DBSs, which have been probed by high-resolution PEI using a third-generation electrospray ionization (ESI)-PES apparatus equipped with a cryogenically cooled Paul trap.^55^ rPES *via* vibrational autodetachment has been shown to be a powerful technique to resolve rich vibrational features, especially for low-frequency and Franck–Condon (FC) inactive vibrational modes, as well as conformation-selective and tautomer-specific spectroscopic information. Additionally, a DBS of the cluster anion C_2_P^–^ was observed, revealing that the weakly dipole-bound electron is not spin-coupled to the core electrons of C_2_P.^56^ In the meantime, DBS resonances of a number of diatomic anions and the associated vibrational autodetachment have also been reported.^57^–^60^

In this perspective, we first discuss the experimental methods in Section 2. We then present the DBSs of C_6_H_5_O^–^ and C_6_H_5_S^–^ in Section 3, illustrating some basic features of the DBSs, such as the small binding energies of the DBSs, structural similarities between an anion in the DBS and its corresponding neutral, and vibrational autodetachment following the Δ*v* = –1 propensity rule. Section 4 presents several applications of rPES in resolving vibrational information by resonant enhancement, from the vibrational origin of the CH_3_COO radical to the low-frequency and FC-inactive vibrational features of the deprotonated uracil radical. Intramolecular inelastic rescattering, which lights up low-frequency FC-inactive vibrational modes, will also be discussed. In Section 5, we present isomer-specific rPES *via* DBSs of two conformers of *m*-HO(C_6_H_4_)O^–^ and two tautomers of deprotonated cytosine anions. The first observation of a quadrupole-bound excited state of cryogenically cooled NC(C_6_H_4_)O^–^ anions will be described in Section 6. Finally, in Section 7, we give a summary and provide some perspectives for the study of noncovalent excited states and rPES of cryogenically cooled anions.

## 2 Experimental methods

This section describes the experimental techniques that we have developed to study excited DBSs of anions. The principle of rPES *via* vibrational autodetachment from DBSs will be discussed, illustrating the differences of rPES from conventional PES. Photodetachment spectroscopy used to search for DBS resonances of anions will be discussed. We will briefly present our current third-generation ESI-PES apparatus,^55^ equipped with a cryogenic Paul trap and high-resolution PEI system, which is critical for the realization of rPES and PDS of cold anions.

### 2.1 Resonant PES *via* vibrational autodetachment and PDS

**Fig. 2:**
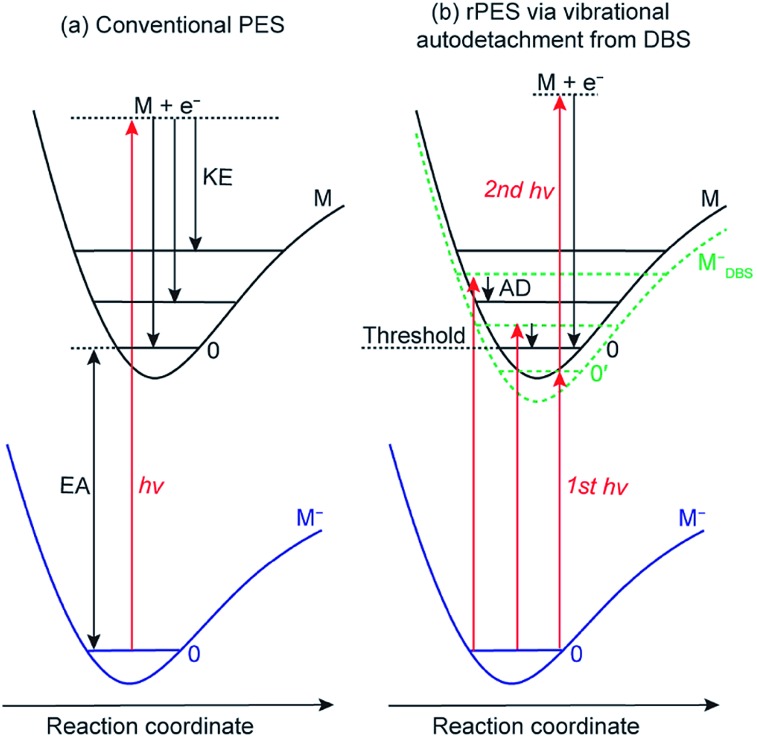
Schematics of (a) non-resonant conventional PES and (b) rPES *via* vibrational autodetachment (AD) from above-threshold vibrational levels of the DBS (the dashed green curve, M_DBS_^–^) and resonant two-photon detachment from the ground vibrational level (0′) of the DBS. EA: electron affinity; KE: kinetic energy; AD: autodetachment.

Conventional anion PES is done at a fixed laser wavelength, as schematically shown in Fig. 2a. A beam of anions (M^–^) is detached by a laser beam. When the laser photon energy (*hv*) exceeds the binding energy of the electron in the anion or the electron affinity (EA) of the corresponding neutral, photoelectrons (e^–^) can be ejected with various kinetic energies (KEs) depending on the resulting final neutral states (M). Conventional PES is governed by the FC principle, only allowing vibrational modes with significant FC factors to be observed, though anomalous PES intensities can be observed in slow-electron velocity-map imaging in certain detachment photon energies^61^,^62^ or due to vibronic coupling^4^,^63^ and excitations to non-valence states.^64^

However, if an excited DBS exists, rPES is possible by tuning the laser wavelength to the DBS vibrational resonances of the anion, as shown in Fig. 2b (M_DBS_^–^). Resonant PES involves two processes. The first is resonant excitation, which has a high absorption cross section, from the anion ground state to the DBS vibrational levels. For below-threshold DBS vibrational levels, a second photon is required to detach the DBS electron. For above-threshold DBS vibrational resonances (aka vibrational Feshbach resonances), vibronic coupling can induce autodetachment from the DBS vibrational levels to neutral levels *via* transfer of vibrational energies to the weakly bound electron. The vibrational autodetachment follows the Δ*v* = –1 propensity rule under the harmonic approximation, which was extended from autoionization of molecular Rydberg states.^42^,^43^ The Δ*v* = –1 propensity rule, which is also related to the fact that the potential energy curve of the DBS and that of the neutral is almost identical (*i.e.*, the DBS electron has little effect on the structure of the neutral core), suggests that only one quantum of vibrational energy is allowed to transfer to the DBS electron (see Fig. 2b). The corresponding neutral final vibrational state in the resonant photoelectron spectrum will display an enhanced intensity in comparison to the vibrational peak in the non-resonant spectrum, due to the large cross section of the resonant excitation process. Hence, rPES is highly non-Franck–Condon.^55^ Because the diffuse dipole-bound electron has little effect on the structure of the neutral core, the geometries of the anion in the DBS and the corresponding neutral are identical, implying that the vibrational frequencies of the DBS are the same as those of the neutral. Therefore, the vibrational frequencies of the corresponding neutral molecules can be obtained by probing the DBS vibrational levels or *vice versa*. It should be pointed that the Δ*v* = –1 propensity rule is derived under the harmonic approximation and can be violated if there are strong anharmonic effects.^42^

DBS vibrational resonances can be searched using photodetachment spectroscopy by scanning a tunable laser across the detachment threshold of an anion while monitoring the total photoelectron yield. When the laser wavelength is in resonance with a DBS vibrational level, the photoelectron yield is enhanced due to autodetachment for above-threshold levels or resonant two-photon detachment for below-threshold vibrational levels (Fig. 2b).

It is interesting to note the differences of DBS vibrational autodetachment from normal vibrational autodetachment involving anions with very low electron binding energies,^65^ first observed for NH^–^.^66^ The vibrational energy in one quantum of NH^–^ is higher than its electron binding energy; hence vibrational excitation to the *v* = 1 vibrational level of NH^–^ can induce electron detachment, *i.e.* vibrational autodetachment. In such a normal vibrational autodetachment, there are usually large FC activities due to the large geometry changes between the anionic initial states and the final neutral states, for which theoretical models have been developed.^43^

### 2.2 The third-generation ESI-PES apparatus

**Fig. 3:**
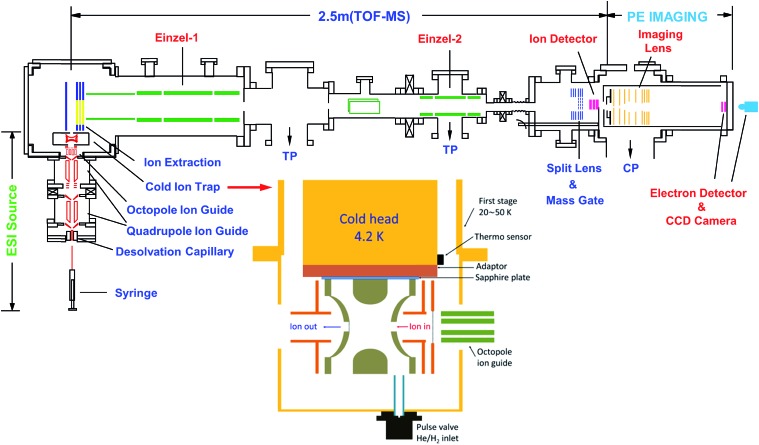
Schematic of the third-generation ESI-PES apparatus, equipped with a cryogenically cooled Paul trap and a high-resolution PEI system. TP: turbomolecular pump. CP: cryopump. Reproduced from ref. 55 with permission from AIP Publishing.

The rPES and PDS experiments were made possible with our third-generation ESI-PES apparatus,^55^ as schematically presented in Fig. 3. It mainly consists of four parts: (1) an ESI source similar to that used in the first ESI-PES apparatus,^67^ (2) a cryogenic Paul trap similar to that developed for the second-generation ESI-PES apparatus,^68^ (3) a TOF mass spectrometer, and (4) a high-resolution PEI analyzer.^69^

Details of the third-generation ESI-PES apparatus and the improvements relative to the first- and second-generation apparatuses have been described previously.^55^ Briefly, anions are produced usually by electrospray ionization of ∼1 mM sample solutions in a mixed solvent of either MeOH/H_2_O or CH_3_CN/H_2_O. Two radio-frequency quadrupole and one octopole ion guides are used to direct anions from the ESI source into a cryogenically cooled Paul trap, which is attached to a helium refrigerator operated at 4.5 K. The anions are cooled *via* collisions with a 1 mTorr He/H_2_ (4/1 in volume) buffer gas, which is shown empirically to exhibit optimal thermal cooling effects.^68^ After being accumulated for 0.1 s and thermally cooled, anions are pulsed out at a 10 Hz repetition rate into the extraction zone of a TOF mass spectrometer. Anions of interest are selected by a mass gate and photodetached in the interaction zone of the PEI lens using a Nd:YAG laser or a tunable dye laser. Photoelectrons are focused by a set of imaging lenses and projected onto a pair of 75 mm diameter micro-channel plates coupled to a phosphor screen and are captured by a charge-coupled device camera. The electron KE resolution is usually 3.8 cm^–1^ for electrons with 55 cm^–1^ energy and about 1.5% (ΔKE/KE) for kinetic energies above 1 eV. The narrowest line width achieved was 1.2 cm^–1^ for 5.2 cm^–1^ electrons.^69^

The third-generation ESI-PES apparatus has allowed the study of weakly bound non-covalent excited states of anions, including both dipole-bound^5^,^41^,^44^–^53^ and quadrupole-bound excited states,^54^ and the development of rPES and PDS for cold anions. In a typical investigation, we first measure non-resonant PE spectra to obtain the detachment threshold of an anion. Then, PDS is used to search for DBS resonances by monitoring the total electron yield as a function of the detachment laser wavelength across the detachment threshold at a step size of 0.1 nm or 0.03 nm. Subsequently, rPES is performed by parking the laser wavelengths at the identified DBS resonances. The enhanced vibrational peaks in rPES can be used to infer the vibrational resonances of the DBS, often assisted by computed vibrational frequencies.

### 2.3 The cryogenically cooled Paul trap

Due to the small binding energies of the DBS electron, it is critical to cool down the anions to low temperatures to allow high-resolution PDS and rPES and facilitate spectral assignments of complex anions by eliminating vibrational hot bands. In 2005, the Wang group developed the first version of a cryogenically cooled Paul trap^68^ and reported the first PES experiment for cold anions from an ESI source.^70^ Different from the cryogenic 22-pole trap,^71^ the cryogenic Paul trap exhibits better 3D confinement of ions, making it more suitable for the subsequent TOF mass selection necessary for the PES and PDS experiments. The current configuration of the cryogenic Paul trap (see inset of Fig. 3) at Brown University features a pulsed buffer gas and a more powerful cryostat.^55^ When the cryostat is operated at 4.5 K, the ion temperature achieved has been estimated to be 30–35 K from simulations of rotational profiles in PDS of several anions.^43^,^44^,^54^ With the complete elimination of vibrational hot bands in the PE spectra of cold C_60_^–^, the most accurate EA of C_60_ was measured to be 2.6835(6) eV, as well as the resolution of sixteen fundamental vibrational frequencies for the C_60_ molecule.^72^

The cryogenic Paul trap has also been adapted by several groups to study cold ions and ionic clusters by vibrational spectroscopy,^73^–^75^ UV photofragmentation,^76^–^79^ UV-UV hole-burning spectroscopy^80^–^82^ and anion slow electron velocity map imaging spectroscopy.^83^

## 3 Basic features of dipole-bound excited states

### 3.1 The observation of an excited DBS in C_6_H_5_O^–^

#### 3.1.1 DBS resonances revealed by photodetachment spectroscopy

**Fig. 4:**
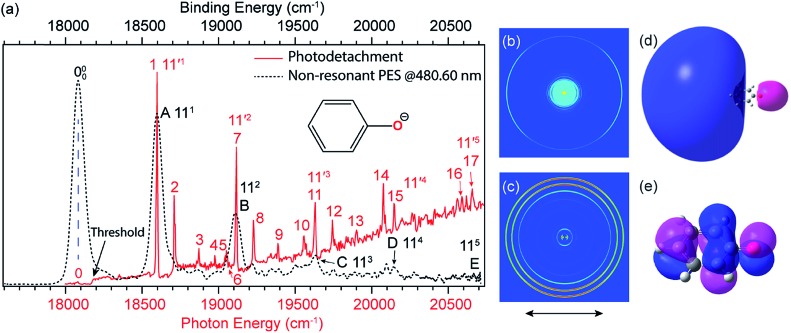
(a) Comparison of the photodetachment spectrum (red solid curve) with the non-resonant PE spectrum at 480.60 nm (black dashed curve) of C_6_H_5_O^–^. The PE spectrum is red-shifted by 97 cm^–1^ to line up peak 000 with peak 0. The vibrational progression of the FC-active mode *ν*_11_ agrees well with each other, indicating that the weakly bound electron in the DBS of C_6_H_5_O^–^ has little effect on the structure of the neutral C_6_H_5_O core. (b) Resonant two-photon image at peak 0. (c) Non-resonant PE image at 480.60 nm. The arrows below the images represent the polarization directions of the laser. (d) The calculated DBS orbital. (e) The HOMO of C_6_H_5_O^–^. Adapted from ref. 51 and 55 with permission from AIP Publishing.

The first anion for which we observed an excited DBS and performed rPES in 2013 was the phenoxide anion (C_6_H_5_O^–^),^41^ because the neutral phenoxy radical C_6_H_5_O possesses a large dipole moment of 4.06 D. Eight DBS vibrational resonances were found manually. Recently, a more complete photodetachment spectrum was obtained for C_6_H_5_O^–^, revealing a total of eighteen vibrational resonances across the detachment threshold at 18 173 cm^–1^ (Fig. 4a, the red solid curve).^41^,^51^ The weak peak 0, below the detachment threshold by 97 cm^–1^, represents the ground vibrational level of the DBS of C_6_H_5_O^–^, which is due to resonant two-photon detachment. Above the threshold, the gradually increasing baseline represents the non-resonant detachment signals. The seventeen peaks (1–17) correspond to excited vibrational levels of the DBS of C_6_H_5_O^–^, *i.e.*, vibrational Feshbach resonances.

#### 3.1.2 The structural similarity between an anion in the DBS and the corresponding neutral

In Fig. 4a, the non-resonant PE spectrum of C_6_H_5_O^–^ at 480.60 nm (black dashed curve) obtained from the PE image in Fig. 4c is overlaid with the photodetachment spectrum (red solid curve). The non-resonant PE spectrum shows the vibrational progression of the most FC-active stretching mode *ν*_11_ up to the fifth quanta,^84^,^85^ represented by peaks A to E. By shifting the PE spectrum by 97 cm^–1^ to line up peak 000 in the PE spectrum with peak 0 in the photodetachment spectrum, we see that the positions and relative intensities of the vibrational progression of mode *ν*_11_ in the PE spectrum and those in the photodetachment spectrum (peaks 1, 7, 11, 15 and 17) are perfectly matched. This comparison vividly demonstrates the structural similarity between the molecular core in the DBS of C_6_H_5_O^–^ and the neutral C_6_H_5_O radical. Since the peak width in the photodetachment spectrum is mainly limited by rotational broadening, the measured frequencies are in general more accurate than those obtained from the PE spectrum, where the spectral resolution depends on the photoelectron kinetic energies. In addition, much richer vibrational features are revealed in the photodetachment spectrum due to the resonant enhancement *via* the DBS. Hence, in comparison with conventional non-resonant PES, rPES in combination with PDS is more powerful to resolve vibrational information for dipolar neutral radicals by probing the DBS resonances.

#### 3.1.3 The s-type orbital of the DBS

By tuning the laser wavelength to the below-threshold peak 0 in Fig. 4a, we obtained the resonant two-photon PE image displaying a *p*-wave angular distribution (the outermost ring in Fig. 4b), which is due to the detachment from the s-type DBS orbital of C_6_H_5_O^–^, as shown in Fig. 4d. In contrast, the non-resonant PE image at 480.60 nm exhibits an s + d perpendicular angular distribution (Fig. 4c), as a result of one-photon detachment from the p-type HOMO orbital of C_6_H_5_O^–^ (Fig. 4e).^41^,^51^

### 3.2 Resonant PE spectra *via* vibrational autodetachment from the DBS of C_6_H_5_O^–^

**Fig. 5:**
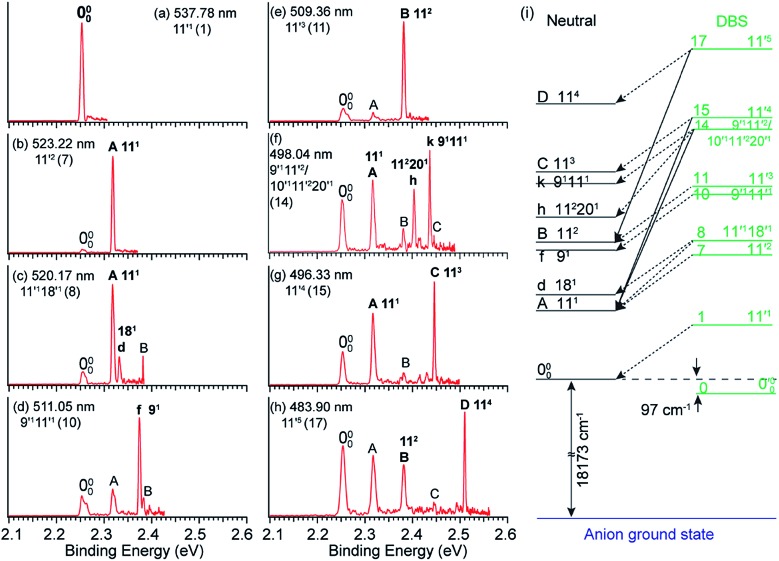
(a–h) High-resolution resonant PE spectra of C_6_H_5_O^–^ at eight different wavelengths, corresponding to the eight DBS resonances (within parentheses) in Fig. 4a. The enhanced peaks *via* vibrational autodetachment from the DBS are labeled in bold face. The assigned vibrational levels of the DBS are given. (i) Schematic energy level diagram for selective vibrational autodetachment from the DBS of C_6_H_5_O^–^ to the neutral vibrational levels of C_6_H_5_O. The detachment threshold (18 173 cm^–1^) and the DBS binding energy (97 cm^–1^) of C_6_H_5_O^–^ are also given. Adapted from ref. 51 with permission from AIP Publishing.

By tuning the detachment laser wavelength to the above-threshold DBS resonances in Fig. 4a, seventeen high-resolution resonant PE spectra were obtained.^41^,^51^ Fig. 5a–h present eight such resonant PE spectra as examples, with photon energies corresponding to DBS resonances 1, 7, 8, 10, 11, 14, 15 and 17. Two detachment channels contribute to the resonant PE spectra: the non-resonant detachment process represented by the continuous baseline in the photodetachment spectrum and the resonantly enhanced vibrational autodetachment *via* the DBS indicated by the sharp peak in the photodetachment spectrum in Fig. 4a. In comparison to the non-resonant PE spectrum at 480.60 nm in Fig. 4a, the resonant PE spectra are highly non-FC with one or more vibrational peaks enhanced due to the mode selectivity and the Δ*v* = –1 propensity rule.

The vibrational DBS resonances consist of single-mode levels 
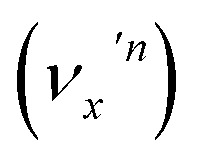
, combinational levels 
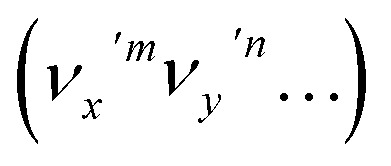
 or nearly degenerate overlapping vibrational levels. Note that a prime is used to designate DBS vibrational modes to distinguish from the corresponding neutral modes. For autodetachment from vibrational levels of a single mode 
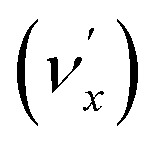
, the *n*th vibrational level of this mode 
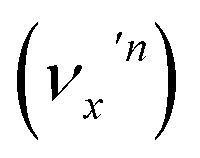
 in the DBS can autodetach to the (*n*–1)th level of the same mode in the neutral 
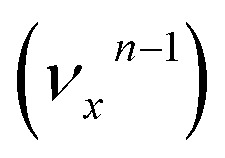
, *i.e.* one quantum of the vibrational energy in mode 
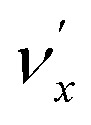
 is transferred to the dipole-bound electron during autodetachment. The resulting final neutral peak in the PE spectrum corresponding to the 
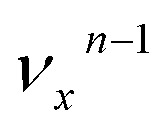
 level will be highly enhanced. For instance, the resonant PE spectra in Fig. 5a, b, e, g and h correspond to excitations to DBS vibrational levels involving mode 
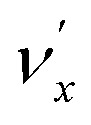
 for *n* = 1 to 5, respectively. Vibrational autodetachment processes from these DBS levels result in significant enhancement of peaks 000, A (11^1^), B (11^2^), C (11^3^) and D (11^4^), respectively, in the resonant PE spectra, following the Δ*v* = –1 propensity rule. In these autodetachment processes, one vibrational quantum of mode 
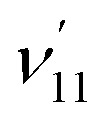
 (519 cm^–1^) is transferred to the DBS electron (BE = 97 cm^–1^), yielding an autodetached electron with a KE of 422 cm^–1^ in all five cases. In addition, peaks A (11^1^) and B (11^2^) are slightly enhanced in Fig. 5g and h, respectively, following a Δ*v* = –3 autodetachment process. This violation of the Δ*v* = –1 propensity rule indicates anharmonicity at higher vibrational levels.^42^

The autodetachment from a combinational vibrational level 
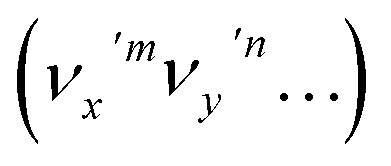
 of the DBS is more complicated. When all the vibrational frequencies of the modes involved are larger than the binding energy of the DBS, both neutral final levels, 
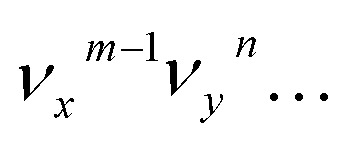
 and 
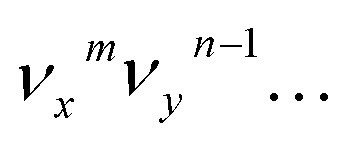
, are expected to be enhanced. Fig. 5c displays such a case, where both peaks A (11^1^) and d (18^1^) are highly enhanced because of autodetachment from the combinational DBS level 11′^1^18′^1^ following the Δ*v* = –1 propensity rule. However, excitation to the DBS combinational level 9′^1^11′^1^ in Fig. 5d only results in strong enhancement of peak f (9^1^), which means that the mode 
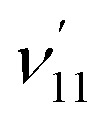
 is more strongly coupled with the dipole-bound electron, indicating mode selectivity in vibronic coupling. Even more complicated cases are those involving autodetachment from overlapping vibrational levels of the DBS, as shown in Fig. 5f, which corresponds to resonant excitation to two nearly degenerate vibrational levels, 9′^1^11′^2^ and 10′^1^11′^2^20′^1^. The enhancement of the two peaks A (11^1^) and k (9^1^11^1^) is due to autodetachment from the DBS level 9′^1^11′^2^, while that of peak h (11^2^20^1^) is due to autodetachment from the 10′^1^11′^2^20′^1^ DBS level. Both mode-selectivity and anharmonic effects are observed. All the discussed autodetachment processes from the DBS vibrational levels to neutral levels are schematically illustrated in Fig. 5i.

### 3.3 Observation of a DBS in C_6_H_5_S^–^

**Fig. 6:**
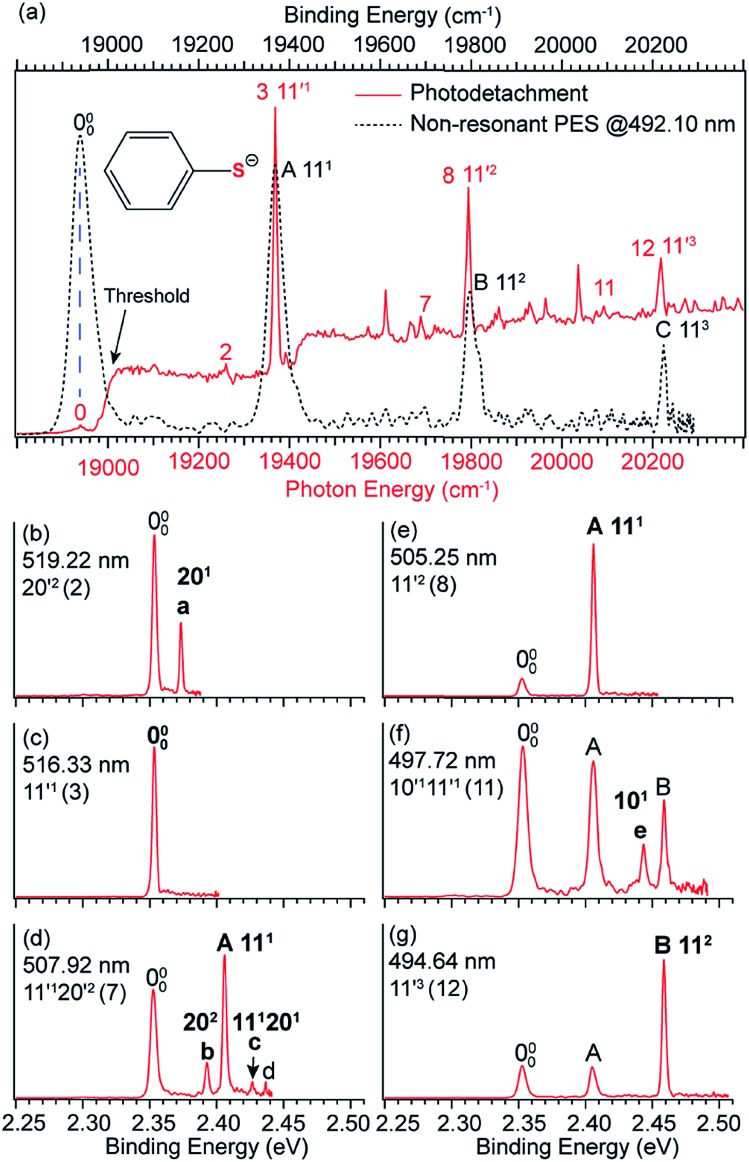
(a) Comparison of the photodetachment spectrum (red solid curve) and the non-resonant PE spectrum at 492.10 nm (black dashed curve) of C_6_H_5_S^–^. The PE spectrum is red-shifted by 39 cm^–1^ to line up peak 000 (the ground vibrational level of neutral C_6_H_5_S) with peak 0 (the ground vibrational level of the DBS of C_6_H_5_S^–^). The vibrational progression of the FC-active mode *ν*_11_ matches well with each other, suggesting the weakly bound electron in the DBS of C_6_H_5_S^–^ has little effect on the neutral core C_6_H_5_S. (b–g) High-resolution resonant PE spectra of C_6_H_5_S^–^ at six different wavelengths. The enhanced peak *via* vibrational autodetachment from the DBS is labeled in bold face. The assigned vibrational levels of the DBS are given. Adapted from ref. 51 with permission from AIP Publishing.

The thiophenoxide anion (C_6_H_5_S^–^) is another relatively simple example that can be used to illuminate the basic features of DBSs and rPES,^51^ as shown in Fig. 6. With a dipole moment of 3.18 D for the thiophenoxy radical (C_6_H_5_S), an excited DBS was observed in the photodetachment spectrum of C_6_H_5_S^–^ (Fig. 6a). The ground vibrational level of the DBS, labeled peak 0 in Fig. 6a, is 39 cm^–1^ below the detachment threshold of C_6_H_5_S^–^ at 18 978 cm^–1^. Similar to the PE spectra of C_6_H_5_O^–^, the non-resonant PE spectra of C_6_H_5_S^–^ were also dominated by the *ν*_11_ vibrational progression.^85^ By aligning peak 0 in the photodetachment spectrum and peak 000 in the non-resonant PE spectrum at 492.10 nm, a perfect agreement is observed for the relative peak positions and intensities of the most FC-active *ν*_11_ vibrational progression, again suggesting little influence of the DBS electron on the neutral C_6_H_5_S core in the DBS. Eleven above-threshold vibrational resonances were observed. Selected high-resolution resonant PE spectra are presented in Fig. 6b–g, which were collected at laser wavelengths corresponding to the selected DBS resonances in Fig. 6a. The highly enhanced peaks a (20^1^) in Fig. 6b, peak 000 in Fig. 6c, peak A (11^1^) in Fig. 6e and peak B (11^2^) in Fig. 6g are due to excitations to DBS vibrational levels 20′^2^, 11′^1^, 11′^2^ and 11′^3^, obeying the Δ*v* = –1 propensity rule for autodetachment. In Fig. 6f, the enhancement of peak e (10^1^) is due to the mode-specific autodetachment from the combinational level 10′^1^11′^1^: strong vibronic coupling is only observed for mode 
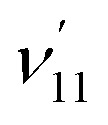
, similar to the case of C_6_H_5_O^–^ (Fig. 5). The resonant PE spectrum in Fig. 6d, corresponding to excitation to the combinational DBS level 11′^1^20′^2^, reveals enhancement of three final vibrational states, labeled b (20^2^), c (11^1^20^1^) and A (11^1^). The autodetachment to peaks b and c follows the Δ*v* = –1 propensity rule, while that to peak A involves Δ*v* = –2 of the lowest frequency bending mode 
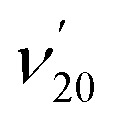
.^51^

## 4 Rich vibrational information from PDS and rPES

The structural similarities between dipole-bound anions and the corresponding neutrals are clearly revealed from the similarities of the vibrational structures of the DBS and the neutrals for the cases of C_6_H_5_O^–^ and C_6_H_5_S^–^, as shown in Fig. 4a and 6a, respectively. These observations confirm spectroscopically that the weakly bound electron in the DBS has little influence on the structure of the neutral core. This observation means that the vibrational frequencies of the neutrals are the same as those in the DBS. Photodetachment spectra often show much richer vibrational features with higher spectral resolution. Resonant PE spectra can “light up” FC-inactive vibrational modes or vibrational transitions with very small FC factors. Hence, the combination of PDS and rPES of cold anions can be a powerful approach to obtain vibrational information for dipolar neutral radicals, inaccessible in other spectroscopic techniques.

### 4.1 Determining accurate EAs *via* resonant enhancement of the 0–0 transition

**Fig. 7:**
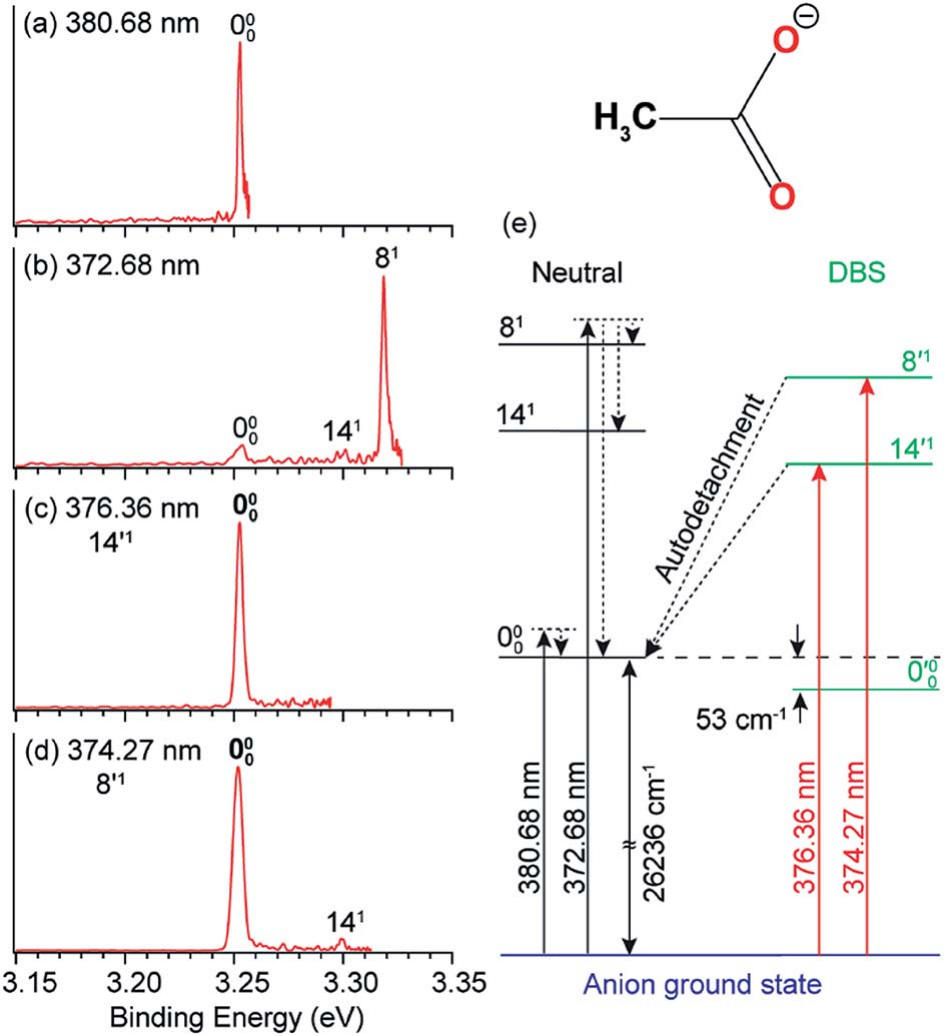
High-resolution non-resonant PE spectra of CH_3_COO^–^ at (a) 380.68 nm and (b) 372.68 nm. High-resolution resonant PE spectra of CH_3_COO^–^ at (c) 376.36 nm and (d) 374.27 nm, which both show the enhancement of peak 000, due to vibrational autodetachment from the fundamental DBS vibrational levels 14′^1^ and 8′^1^ of CH_3_COO^–^, respectively. (e) Schematic energy level diagram showing direct detachment in the non-resonant spectra and autodetachment from the DBS vibrational levels. The detachment threshold (26 236 cm^–1^) and the DBS binding energy (53 cm^–1^) of CH_3_COO^–^ are also given. Adapted from ref. 45 with permission from AIP publishing.

In anion PES, the 0–0 transition defines the EA of the corresponding neutral species. However, for large geometry changes between the anion and neutral, the FC factor for the 0–0 transition may be extremely weak, making it difficult to be observed and identified in conventional PES. According to the Δ*v* = –1 propensity rule, autodetachment from fundamental DBS vibrational levels can result in considerable resonant enhancement of the 0–0 detachment transition. This resonant enhancement can be very valuable in the assignment of the 0–0 transition and in determining the EA of neutrals with large geometry changes in anion PES. For example, the photodetachment from CH_3_COO^–^ results in a large reduction of the ∠O–C–O angle by ∼20° in the neutral CH_3_COO radical,^86^ which results in a very weak FC factor for the 0–0 transition. If the anions are vibrationally hot, the weak peak 000 would be buried in the vibrational hot bands, making it challenging to accurately determine the EA of CH_3_COO.^87^,^88^ With the third generation ESI-PES apparatus, a high-resolution non-resonant spectrum of cold CH_3_COO^–^ at 372.68 nm revealed a very weak feature for the 000 transition and two vibrational peaks, 14^1^ and 8^1^ (Fig. 7b).^45^ When tuning the laser wavelength near the detachment threshold at 380.68 nm, peak 000 is better measured, giving rise to an accurate EA of 26 236 ± 8 cm^–1^ for CH_3_COO (Fig. 7a). However, the non-resonant spectrum required a very long time for signal accumulation due to the poor FC factor.^45^

Because the CH_3_COO radical has a dipole moment of 3.47 D (Fig. 1), CH_3_COO^–^ was found to support a DBS with a binding energy of 53 cm^–1^.^45^ Even though the FC factor is small for the 0–0 transition, there are strong FC activities to vibrationally excited levels in both the PE spectra and the photodetachment spectrum. When the detachment laser was tuned to the DBS vibrational resonances corresponding to the 14′^1^ and 8′^1^ vibrational levels, two resonant PE spectra (Fig. 7c and d) were obtained, exhibiting significant enhancement for peak 000 and confirming its origin as the 0–0 transition. The relevant non-resonant and resonant detachment transitions are shown schematically in the energy level diagram in Fig. 7e.

### 4.2 Observation of Franck–Condon-inactive low-frequency vibrational modes

Conventional non-resonant PES is governed by the FC principle, which means that only FC-allowed or totally symmetric vibrational modes can be observed usually. However, rPES involving optical excitations to DBS levels can “light up” FC-inactive modes due to the large optical absorption cross sections relative to non-resonant photodetachment processes. For example, the lowest-frequency symmetry-forbidden and FC-inactive *ν*_20_ bending mode of C_6_H_5_S, absent in the non-resonant spectra, is revealed prominently in the resonant PE spectrum in Fig. 6b, when the 20′^2^ DBS vibrational level is excited.^51^ The combination of PDS and rPES has been shown to be particularly powerful to allow low frequency and FC-inactive modes to be observed.

One of the most prominent examples is the deprotonated uracil radical ([U–H] or C_4_N_2_O_2_H_3_),^5^,^44^ which has a total of twenty seven fundamental vibrational modes (Table 1), including nineteen in-plane vibrational modes (A′) and eight out-of-plane modes (A′′). With a dipole moment of 3.22 D for the neutral core, the deprotonated uracil anion ([U–H]^–^, Fig. 1) was found to possess a DBS below the detachment threshold by 146 cm^–1^. By scanning the laser wavelength up to ∼1700 cm^–1^ above the threshold, a total of forty-six DBS vibrational levels were observed.^5^,^44^ The combination of PDS and rPES allowed fundamental vibrational frequencies for twenty-one modes to be observed, including seven out of the eight symmetry-forbidden out-of-plane modes, as shown in Table 1. Even more vibrational modes could have been observed if we were to scan the laser to higher excitation energies to probe more DBS resonances.

**Table 1:** Comparison of theoretical and experimental vibrational frequencies (in cm^–1^) of the deprotonated uracil radical ([U–H]) measured from PDS and rPES. Reproduced from ref. 5 with permission from Elsevier

Mode	Symmetry	Theo. freq.	Exp. freq.
*ν*_1_	A′	3581	
*ν*_2_		3222	
*ν*_3_		3145	
*ν*_4_		1713	1705
*ν*_5_		1694	1672
*ν*_6_		1469	1451
*ν*_7_		1437	
*ν*_8_		1411	
*ν*_9_		1342	1316
*ν*_10_		1301	1285
*ν*_11_		1186	1190
*ν*_12_		1082	1057
*ν*_13_		982	970
*ν*_14_		920	910
*ν*_15_		757	753
*ν*_16_		583	577
*ν*_17_		545	540
*ν*_18_		501	492
*ν*_19_		392	389
*ν*_20_	A′′	980	
*ν*_21_		803	804
*ν*_22_		734	727
*ν*_23_		684	666
*ν*_24_		633	615
*ν*_25_		357	360
*ν*_26_		152	150
*ν*_27_		113	113

### 4.3 Intramolecular inelastic scattering

**Fig. 8:**
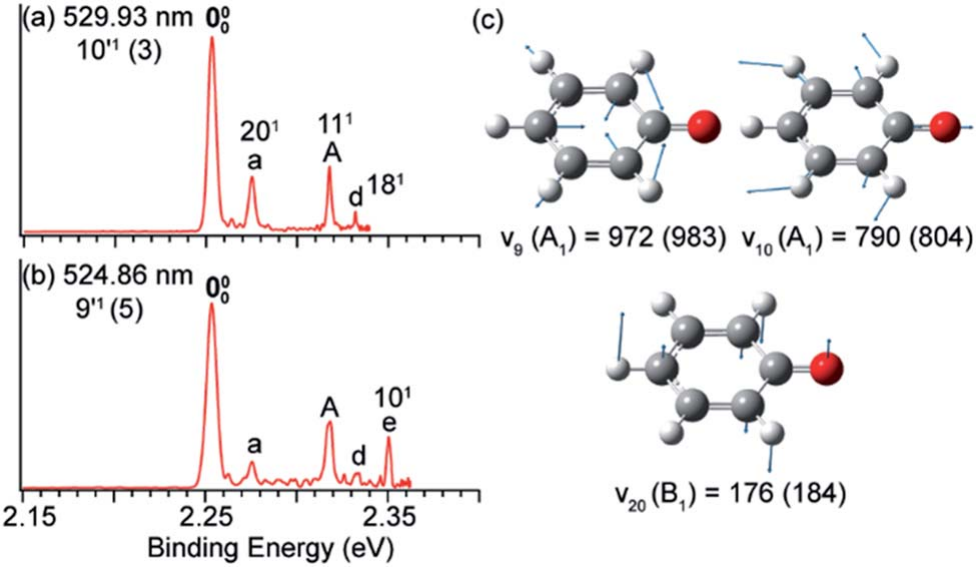
Resonant PE spectra of C_6_H_5_O^–^ at (a) 529.93 nm and (b) 524.86 nm, corresponding to the DBS vibrational levels 10′^1^ and 9′^1^, respectively. (c) The three relevant vibrational modes of neutral C_6_H_5_O. The measured frequencies and the calculated values (within parentheses) are given in cm^–1^. Adapted from ref. 51 with permission from AIP publishing.

**Fig. 9:**
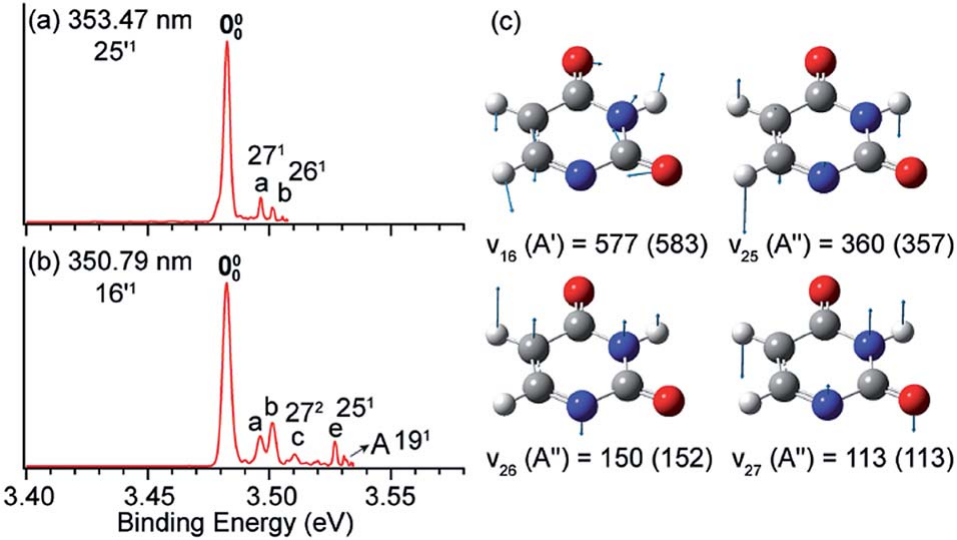
Resonant PE spectra of [U–H]^–^ at (a) 353.47 nm and (b) 350.79 nm, corresponding to the DBS vibrational levels 25′^1^ and 16′^1^, respectively. (c) The four relevant vibrational modes of the neutral radical [U–H]. The measured frequencies and the calculated values (within parentheses) are given in cm^–1^. Adapted from ref. 5 with permission from Elsevier.

In Fig. 8a and b, peak 000 is enhanced due to the Δ*v* = –1 autodetachment from the 10′^1^ and 9′^1^ vibrational levels of the DBS of C_6_H_5_O^–^, corresponding to peaks 3 and 5, respectively, in the photodetachment spectrum in Fig. 4a.^51^ Peak a corresponds to the out-of-plane *ν*_20_ mode (Fig. 8c), which is symmetry-forbidden, but it is present in the resonant PE spectra quite prominently. In the same way, when exciting to the vibrational levels 25′^1^ (Fig. 9a) and 16′^1^ (Fig. 9b) of the DBS of [U–H]^–^, the enhancement of peak 000 following the Δ*v* = –1 autodetachment is accompanied with prominent excitations of several low-frequency modes (Fig. 9c), peaks a (27^1^), b (26^1^), c (27^2^), and e (25^1^), which are symmetry-forbidden in the non-resonant spectra.^5^

Vibronic coupling or Herzberg–Teller coupling^4^,^63^,^72^,^89^ has been previously invoked to explain the observations of FC-inactive vibrational modes or anomalous vibrational intensities in non-resonant PES. While we cannot rule out the effects of vibronic coupling for the appearance of the low-frequency FC-inactive and symmetry-forbidden bending modes in the resonant PE spectra shown in Fig. 8 and 9, a more interesting possibility is intramolecular inelastic rescattering due to the interactions of the autodetached outgoing electron with the neutral core. The rescattering process is possible because the DBS electron is highly diffuse and far away from the neutral core. Hence, there is a finite probability for the outgoing electron to interact inelastically with the neutral core because of exciting low-frequency vibrational modes, akin to processes in electron energy loss spectroscopy.^90^,^91^ Take Fig. 9b as an example: autodetachment from the DBS vibrational level 16′^1^ (*ν*_16_ = 577 cm^–1^, Table 1) of [U–H]^–^ yields an outgoing photoelectron with a kinetic energy of 431 cm^–1^ by subtracting the 146 cm^–1^ binding energy of the DBS. Because of the highly diffuse DBS orbital, it is conceivable that the autodetached electron may have finite probabilities to interact with the neutral core (*i.e.* half-collision or intramolecular rescattering) and lose energies to the bending modes *ν*_27_ (113 cm^–1^), *ν*_26_ (150 cm^–1^), and *ν*_25_ (360 cm^–1^), corresponding to peaks a, b and e, respectively. We have observed especially pronounced rescattering effects for autodetachment from the 16′^1^ DBS level of [U–H]^–^. This observation is not well understood currently and it would deserve some careful theoretical consideration.

## 5 Conformer-selective rPES *via* DBSs

One interesting application of rPES is to obtain conformer-selective spectroscopic information for dipolar species because different conformers have different DBSs. If multiple conformers are present in the ion beam, a non-resonant PE spectrum would be a mixture of the two species. However, enhancement of vibrational features for a specific conformer or conformer-selective rPES can be achieved when the detachment laser is tuned to the DBS vibrational levels of a specific conformer.^48^,^49^,^52^

### 5.1 Conformer-selective rPES *via* the DBS of *m*-HO(C_6_H_4_)O^–^

**Fig. 10:**
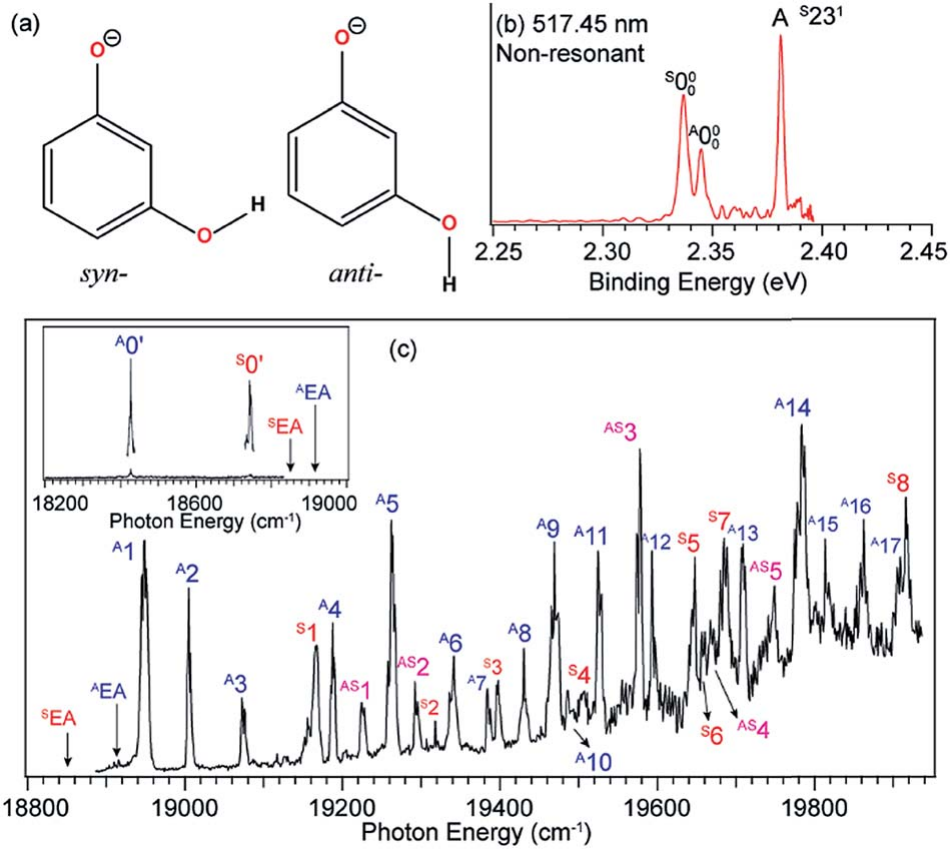
(a) Molecular structures of *syn*- and *anti-m*-HO(C_6_H_4_)O^–^. (b) Non-resonant PE spectrum of *m*-HO(C_6_H_4_)O^–^ at 517.45 nm. The superscripts “S” and “A” in the labels refer to the *syn*- and *anti*-conformers, respectively. (c) The photodetachment spectrum of *m*-HO(C_6_H_4_)O^–^. The two arrows (^A^EA and ^S^EA) indicate the detachment thresholds for *anti*- and *syn-m*-HO(C_6_H_4_)O^–^, respectively. The two peaks, labeled ^A^0′ and ^S^0′ (inset), represent the respective DBS ground states of *anti*- and *syn-m*-HO(C_6_H_4_)O^–^. Peaks ^A^1–^A^17 are due to DBS vibrational levels of *anti-m*-HO(C_6_H_4_)O^–^, peaks ^S^1–^S^8 are due to *syn-m*-HO(C_6_H_4_)O^–^, and peaks ^AS^1–^AS^5 are due to overlapping levels of both conformers. Adapted from ref. 49 with permission from AIP Publishing.

The 3-hydroxyphenoxide anion has two nearly degenerate conformers, *syn*- and *anti-m*-HO(C_6_H_4_)O^–^, due to the different orientations of the hydrogen atom on the –OH group, as shown in Fig. 10a. The non-resonant PE spectrum at 517.45 nm (Fig. 10b) at low temperatures exhibits detachment transitions from both conformers, labeled ^S^000, ^A^000, and A (^S^23^1^).^48^,^49^ Note that the superscripts “A” and “S” designate the *anti*- and *syn*-conformations, respectively. Peaks ^S^000 and ^A^000, with binding energies of 18 850 cm^–1^ and 18 917 cm^–1^, represent the EAs of the *syn*- and *anti-m*-HO(C_6_H_4_)O radicals, respectively. Peak A is a vibrational feature of mode *ν*_23_ of *syn-m*-HO(C_6_H_4_)O. With dipole moments of 3.10 D and 5.34 D for the *syn*- and *anti*-radicals (Fig. 1), respectively, both the anionic conformers are able to support a DBS, as shown in the photodetachment spectrum in Fig. 10c. The weak peaks ^S^0′ and ^A^0′, below the respective detachment thresholds by 104 cm^–1^ and 490 cm^–1^ (inset in Fig. 10c), represent the ground vibrational levels of the DBS for *syn*- and *anti-m*-HO(C_6_H_4_)O^–^, respectively. The larger DBS binding energy of *anti-m*-HO(C_6_H_4_)O^–^ is consistent with the larger dipole moment of its neutral radical. A complicated detachment spectrum was observed with DBS resonances from both conformers: peaks ^A^1–^A^17 are due to *anti-m*-HO(C_6_H_4_)O^–^, peaks ^S^1–^S^8 are due to *syn-m*-HO(C_6_H_4_)O^–^, and peaks ^AS^1–^AS^5 are due to overlapping vibrational levels of both conformers.

**Fig. 11:**
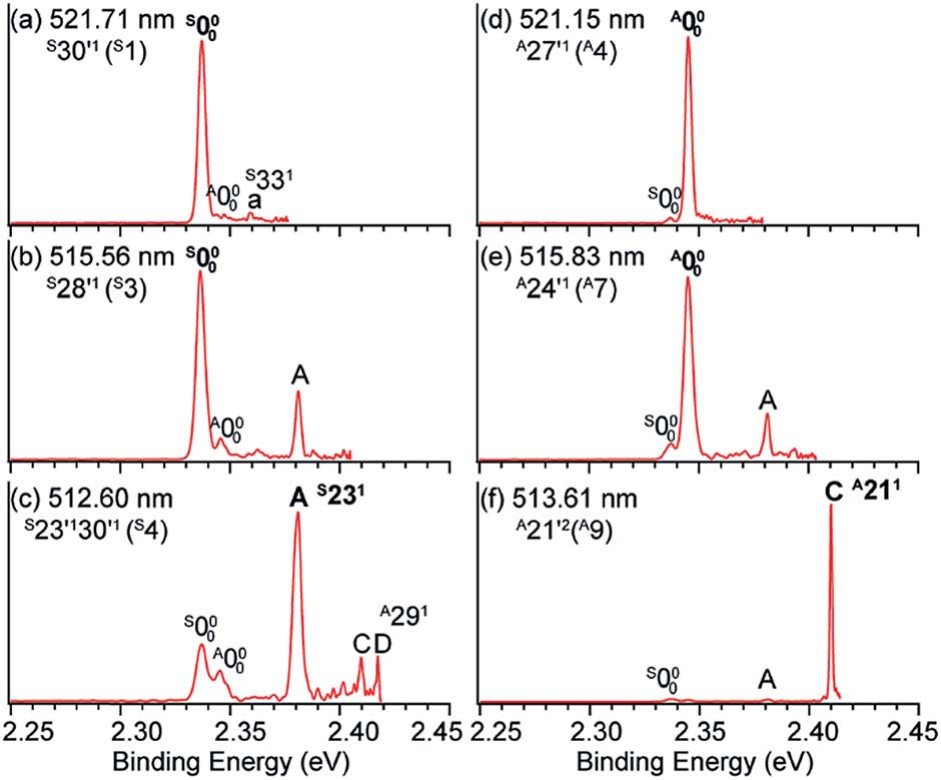
Conformer-specific resonant PE spectra of *syn-m*-HO(C_6_H_4_)O^–^ at (a) 521.71 nm, (b) 515.56 nm, and (c) 512.60 nm and *anti-m*-HO(C_6_H_4_)O^–^ at (d) 521.15 nm, (e) 515.83 nm, and (f) 513.61 nm. The peak numbers within parentheses correspond to the DBS resonances in Fig. 10c. The enhanced peaks *via* vibrational autodetachment from the DBS are labeled in bold face. Adapted from ref. 49 with permission from AIP Publishing.

Hence, by tuning the detachment laser to DBS levels of specific conformers, conformer-selective resonant PE spectra can be obtained. When the detachment laser is tuned to the DBS vibrational levels ^S^30′^1^ and ^S^28′^1^ of *syn-m*-HO(C_6_H_4_)O^–^, the resonant PE spectra display major enhancement of the ^S^000 peak as shown in Fig. 11a and b, where the ^A^000 peak is negligible. When the laser is tuned to the DBS levels ^A^27′^1^ and ^A^24′^1^ of *anti-m*-HO(C_6_H_4_)O^–^, the ^A^000 peak is greatly enhanced as shown in Fig. 11d and e, whereas the ^S^000 peak becomes negligible. In Fig. 11c and f, peaks A (^S^23^1^) and C (^A^21^1^) are enhanced due to autodetachment from DBS levels ^S^23′^1^30′^1^ and ^A^21′^2^, respectively. Such conformer-selective resonant PE spectra have been obtained from every DBS resonance in Fig. 10c, except the five overlapping resonances of the two conformers.^49^

### 5.2 Tautomer-specific rPES *via* the DBS of [Cy-H]^–^

**Fig. 12:**
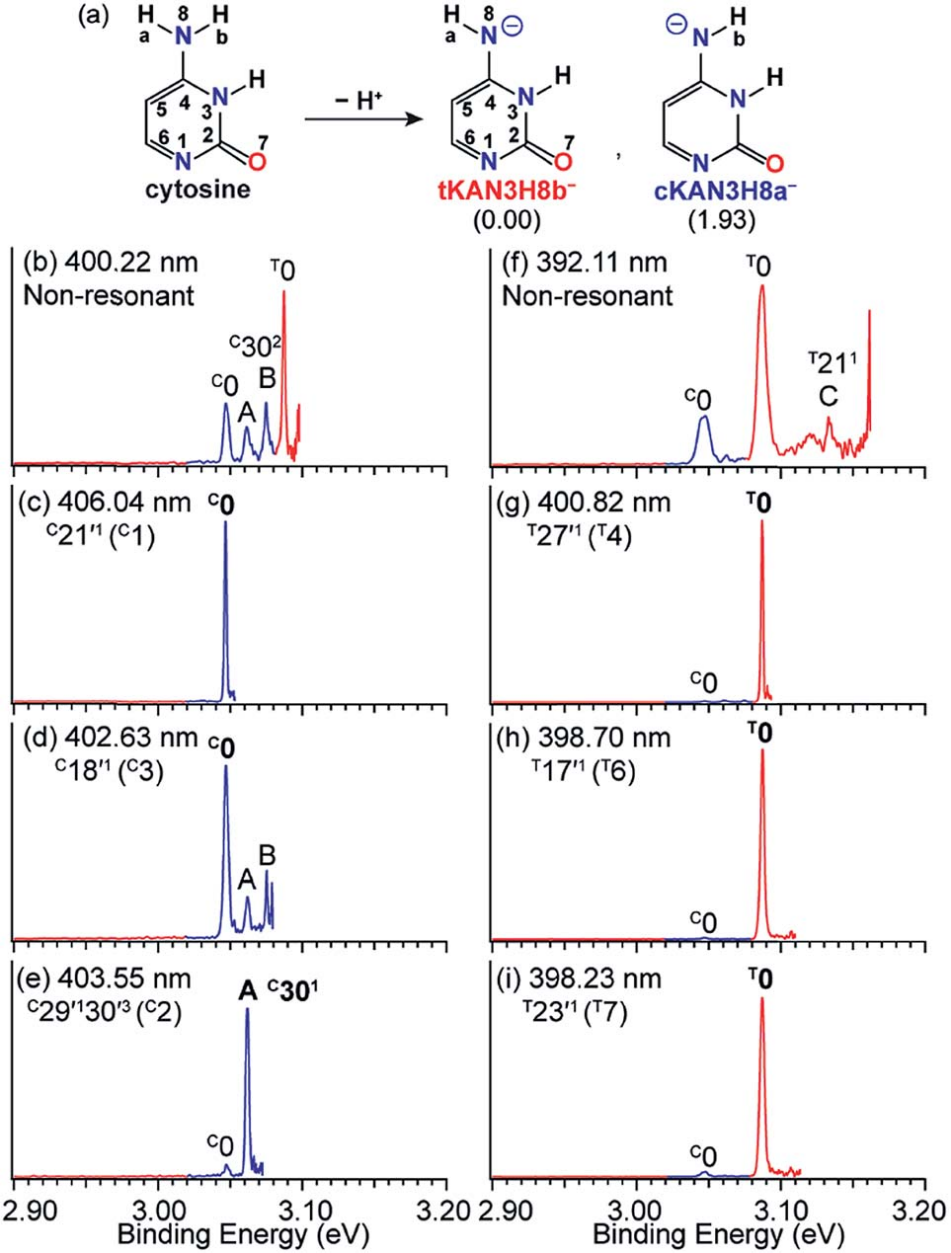
(a) The two most stable anionic tautomers, tKAN3H8b^–^ and cKAN3H8a^–^ upon deprotonation of cytosine. The numbers within parentheses are the relative energies given in kcal mol^–1^. (b and f) Non-resonant PE spectra of [Cy-H]^–^. (c–e) and (g–i) Tautomer-specific resonant PE spectra of tKAN3H8b^–^ and cKAN3H8a^–^. Peaks labeled in bold face indicate the enhanced final neutral vibrational levels due to autodetachment from the DBS. Adapted from ref. 52 with permission. Copyright Wiley-VCH Verlag GmbH & Co. KGaA.

Tautomerism of nucleic acid bases plays an important role in the structure and function of DNA. For example, the deprotonation of cytosine can produce many tautomeric negative ions ([Cy-H]^–^).^92^ Previous calculations^93^ found that the two most stable deprotonated anions in the gas phase are tKAN3H8b^–^ and cKAN3H8a^–^ (Fig. 12a) by deprotonation of H_b_ and H_a_, respectively. The tKAN3H8b^–^ anion was calculated recently to be more stable by 1.93 kcal mol^–1^.^52^ In Fig. 12f, the non-resonant PE spectrum of [Cy-H]^–^ at 392.11 nm reveals three major peaks, labeled ^C^0, ^T^0 and C (^T^21^1^).^52^ Note that the superscripts “C” and “T” designate the tautomers of cKAN3H8a^–^ and tKAN3H8b^–^. Peaks ^C^0 and ^T^0 represent the 0–0 detachment transitions and yield the EAs of cKAN3H8a and tKAN3H8b to be 3.047 eV and 3.087 eV, respectively, which are in excellent agreement with the calculated EAs.^52^ The higher intensity of peak ^T^0 than ^C^0 is consistent with the computed relative stabilities of the two anionic tautomers. Hence, both tautomers are present experimentally even under our low temperature conditions. At 400.22 nm (Fig. 12b), two more vibrational features of cKAN3H8a, labeled A (^C^30^1^) and B (^C^30^2^), are observed.

The cKAN3H8a and tKAN3H8b radicals are calculated to have dipole moments of 3.35 D and 5.55 D (Fig. 1), respectively, which are large enough to support a DBS for the corresponding anions. Distinct DBS vibrational resonances have been observed in the photodetachment spectra of tKAN3H8b^–^ and cKAN3H8a^–^, allowing tautomer-specific resonant PE spectra to be obtained, as presented in Fig. 12c–e and g–i. The resonant PE spectra in Fig. 12c and d show enhancement of peak ^C^0, due to autodetachment from the ^C^21′^1^ and ^C^18′^1^ DBS vibrational levels of cKAN3H8a^–^, respectively. The highly enhanced peak A (^C^30^1^) in Fig. 12e is due to resonant excitation to the ^C^29′^1^30′^3^ DBS level followed by Δ*v* = –3 autodetachment, breaking the Δ*v* = –1 propensity rule. The resonant PE spectra in Fig. 12g–i all display a strongly enhanced ^T^0 peak due to autodetachment from DBS vibrational levels ^T^27′^1^, ^T^17′^1^ and ^T^23′^1^ of tKAN3H8b^–^, respectively, whereas the ^C^0 peak from the cKAN3H8a^–^ tautomer is negligible.

## 6 Quadrupole-bound excited states in NC(C_6_H_4_)O^–^

Long-range charge–quadrupole interactions can form quadrupole-bound anions (QBAs).^3^,^94^,^95^ The rhombic (BeO)_2_^–^ cluster was first suggested to be a QBA.^96^ However, PES of a similar (MgO)_2_^–^ cluster showed a relatively high electron binding energy,^97^ suggesting that this cluster anion should probably be considered as a valence-bound anion.^3^ Similar rhombic alkali-halide dimers, such as (NaCl)_2_ and (KCl)_2_, and a series of complex organic molecules with vanishing dipole moments but large quadrupole moments have also been proposed to form QBAs.^98^–^100^ Experimental studies of electron binding to quadrupolar molecules have been scarce.^101^,^102^ A more recent example of QBAs was from Rydberg electron transfer to the *trans*-isomer of 1,4-dicyanocyclohexane, which has no dipole moment.^103^ A valence-bound anion with a non-polar core may support the excited quadrupole-bound state (QBS) just below the electron detachment threshold, if the neutral core possesses a large quadrupole moment.

**Fig. 13:**
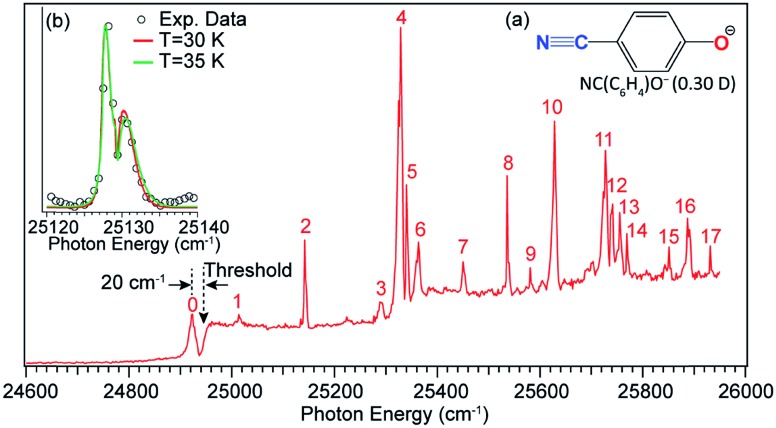
Photodetachment spectrum of NC(C_6_H_4_)O^–^. The dashed arrow at 24 927 cm^–1^ indicates the detachment threshold of NC(C_6_H_4_)O^–^. Peak 0, below the threshold by 20 cm^–1^, represents the vibrational ground state of the QBS due to two-photon detachment. Peaks 1–17 are vibrational resonances of the QBS of NC(C_6_H_4_)O^–^. Inset (a): molecular structure of NC(C_6_H_4_)O^–^ and the dipole moment of the neutral NC(C_6_H_4_)O radical. Inset (b): high-resolution scan of peak 2 revealing the rotational profile, which is fitted by the rotational simulations at 30 K and 35 K. Adapted with permission from ref. 54. Copyright 2017 American Physical Society.

The 4-cyanophenoxide anion [NC(C_6_H_4_)O^–^, see Fig. 13 inset (a)] was found to be a good candidate in the search for the first excited QBS.^54^ The neutral radical, NC(C_6_H_4_)O, has two dipolar centers (–C≡N and C–O) in the opposite direction, resulting in a small dipole moment of 0.30 D but a large quadrupole moment (traceless quadrupole moment: *Q*_*xx*_ = 5.4, *Q*_*yy*_ = 15.1, *Q*_*zz*_ = –20.5 D Å). The dipole moment is much smaller than the 2.5 D critical value to form an excited DBS, but the large quadrupole moment may allow a QBS. Photodetachment spectroscopy of NC(C_6_H_4_)O^–^ indeed revealed many resonances across the detachment threshold at 24 927 cm^–1^, as presented in Fig. 13. A broad peak labeled 0 is observed, 20 cm^–1^ below the detachment threshold, due to resonant two-photon detachment. Since NC(C_6_H_4_)O^–^ cannot support a DBS, peak 0 should represent the ground vibrational level of the QBS. The continuous baseline above the threshold represents the non-resonant detachment signals, while the seventeen peaks, labeled 1–17, are vibrational resonances of the QBS of NC(C_6_H_4_)O^–^. Inset (b) of Fig. 13 shows a high-resolution scan of resonant peak 2, revealing a rotational profile. Rotational simulations yield a rotational temperature between 30 and 35 K for the cryogenically cooled NC(C_6_H_4_)O^–^ anion, consistent with previous results.^5^,^44^,^45^ The vibrational autodetachment processes *via* the QBS are found to be the same as those *via* the DBS, following the Δ*v* = –1 propensity rule. Seventeen resonant PE spectra were obtained, which together with the photodetachment spectrum yielded ten fundamental vibrational frequencies for the NC(C_6_H_4_)O radical.^54^

## 7 Conclusions and outlook

The development of the third-generation ESI-PES with a cryogenically cooled Paul trap and a high-resolution photoelectron imaging system has made it possible to conduct high-resolution spectroscopic investigations of solution-phase anions in the gas phase and, in particular, has enabled high-resolution studies of anions with noncovalent excited states (DBSs or QBSs). Photodetachment spectroscopy has been used to search for both dipole- and quadrupole-bound excited states of cryogenically cooled anions. Resonant PES has been performed *via* autodetachment from above-threshold vibrational levels of non-covalent excited states, resulting in highly non-Franck–Condon PE spectra and rich vibrational information. The weakly-bound electron in the non-covalent excited states has been shown spectroscopically to have negligible effect on the neutral core. Hence, PDS and rPES can be combined to yield much richer vibrational information for the corresponding neutral radicals not accessible by other spectroscopic means. The resonant enhancement of the 0–0 transition in rPES *via* autodetachment from fundamental vibrational levels of DBSs or QBSs allows accurate measurements of EAs for neutrals which have large geometry changes from the corresponding anions. Low-frequency FC-inactive or symmetry-forbidden vibrational modes of various radical species have been observed in rPES. Both mode-selectivity and intramolecular inelastic rescattering have been observed for vibrational autodetachment *via* DBSs. Polar anions with multiple conformers or energetically close tautomers have different DBSs, which allow conformer- or tautomer-specific resonant PE spectra to be realized.

There are many interesting questions that can be investigated using PDS and rPES, as well as experimental challenges. For all the anionic systems we have studied (Fig. 1), the smallest dipole moment (3.03 D) occurs for the neutral core of *o*-HO(C_6_H_4_)O^–^, which gives the smallest DBS binding energy of 25 cm^–1^,^46^ while the deprotonated 4,4′-biphenol anion [HO(C_6_H_4_)_2_O^–^] has a large neutral core dipole moment of 6.35 D with a DBS binding energy of 659 cm^–1^.^54^ The DBS binding energy generally increases with the magnitude of the dipole moment. But there are exceptions. For example, the phenoxy radical has a dipole moment of 4.06 D and the DBS of C_6_H_5_O^–^ is found to have a binding energy of 97 cm^–1^. Yet the DBS in *syn-m*-HO(C_6_H_4_)O^–^ has a larger binding energy of 104 cm^–1^ while its neutral core has a smaller dipole moment of 3.10 D. This indicates that molecular structures and polarizability play important roles in the electron binding in DBSs. Thus, it would be interesting to investigate how the DBS binding energies depend on the magnitude of the dipole moment for different classes of molecular species^1^,^32^ and if the 2.5 D empirical critical dipole moment holds for dipole-bound excited states.^104^

Another interesting question is if it would be possible for an anion to support a second bound DBS below the detachment threshold? If so, what would be the critical dipole moment required for the neutral core? Early theoretical studies of a fixed dipolar system predicted that a critical dipole moment of 9.64 D was required to support a second bound DBS.^105^ A much lower critical value of 4.5 D was proposed later when considering rotational effects.^106^ However, in our attempt to search for a second DBS in the deprotonated 2-hydroxypyrimidine anion (C_4_H_3_N_2_O^–^, Fig. 1),^50^ which has a core dipole moment of 6.15 D, we did not find experimental evidence for a second DBS below the detachment threshold, even though a relatively large binding energy of 598 cm^–1^ was observed for the DBS. The neutral core of HO(C_6_H_4_)_2_O^–^ has the largest dipole moment of 6.35 D among all the anions that we have investigated thus far (Fig. 1). We have recently observed a DBS for this anion with a binding energy of 659 cm^–1^ below the threshold.^53^ But no evidence of a second bound DBS was observed.

Resonant PES *via* noncovalent excited states is unique to resolve low-frequency vibrational modes for the relatively small aromatic systems shown in Fig. 1. There are huge opportunities to extend these studies to larger and more complex systems, such as polyaromatic hydrocarbon-related molecules or molecular clusters. However, because of the high density of low frequency modes with such complicated systems, much better cooling of the initial anions would be required. Another promising direction would be a direct experimental probe of the dynamics of the vibronic coupling in the noncovalent excited states and the autodetachment processes. For the below-threshold DBS resonances, the lifetime and decay dynamics may be insightful to understand the long-range charge–dipole interactions and the transition from the DBS to valence-bound states. Above the threshold, the dynamics and mechanism of vibrational autodetachment should be quite exciting and may be directly investigated by pump-probe experiments. Questions about vibrational mode-selectivity and intramolecular rescattering during vibrationally induced autodetachment may be directly addressed. Such studies along with the results presented here would provide stringent benchmarks to test theories of vibronic coupling.

## Conflicts of interest

There are no conflicts to declare.
